# Editorial of Special Issue “Fibrodysplasia Ossificans Progressiva: Studies on Disease Mechanism towards Novel Therapeutic Approaches”

**DOI:** 10.3390/biomedicines10010140

**Published:** 2022-01-10

**Authors:** Roberto Ravazzolo

**Affiliations:** Department of Neurosciences, Rehabilitation, Ophthalmology, Genetics, Maternal and Child Sciences (DiNOGMI), University of Genova, 16100 Genova, Italy; rravazzo@gmail.com

**Keywords:** fibrodysplasia ossificans progressiva, heterotopic ossification, disease mechanism, therapeutic strategy

## Abstract

The Special Issue on “*Fibrodysplasia Ossificans Progressiva: Studies on Disease Mechanism towards Novel Therapeutic Approaches*” has published interesting and useful review articles and original experimental articles on fibrodysplasia ossificans progressiva (FOP), a very rare genetic disorder for which much effort is being devoted to search for a cure. In this editorial, I briefly cite the essential content of all the published articles.

## 1. Review Articles

Kitoh [1] has presented a comprehensive review on clinical aspects of FOP and interesting highlights on the interaction of the filamin B gene with the Runx2-TGF beta-SMADs pathway and the role of Indian Hedgehog in the pathogenesis of osteochondromas. The article by Khan et al. [2] reviews non-skeletal manifestations of FOP, in particular cardiopulmonary and neurologic ones, recalling the role of the ACVR1/ALK2 gene in cardiac and neurological development, the role of BMP signaling in neuropathic pain and in the myelination process. Cappato et al. [3] have reviewed the acquired and genetic forms of heterotopic ossification by providing a comparison between the human condition and the corresponding animal models, with a critical view on strengths and critical points of these models. Ventura et al. [4] have presented an extensive in-depth review of existing drugs, either already approved or under advanced investigation, that represent a useful repository of compounds to be tested in pre-clinical models of FOP. The authors very convincingly underline how repurposed drugs represent a convenient approach to speed up and lower the cost of clinical experimentation. Katagiri et al. [5], in addition to reviewing the pathogenic role of the mutated ACVR1 gene in FOP, expand their review to other disorders affecting the bone, brain, heart, and skin were found to be associated with ALK2/ACVR1, thus providing new knowledge on this receptor and its related pathogenic mechanisms. Ravazzolo and Bocciardi [6] have explored the reason for the high recurrence rate (more than 95% of FOP patients) of a single mutation in the ACVR1 gene and how the expression of ACVR1 can be modulated by cis-acting variants in the surrounding genomic region of the gene in human chromosome 2q, and have discussed the general issue of genetic modifiers in FOP.

## 2. Experimental Articles

Williams et al. [7] have described the binding of FKBP12.6 to type I receptors using purified recombinant proteins and the structure of ALK2 in complex with FKBP12.6, discussing how the binding of BMP/TGF-beta receptors to FKBPs is of interest not only for the understanding of disease mechanisms, but also to give rise to new drug development strategies. Schoenmaker et al. [8] have investigated how treatment with Activin A induces transcriptomic effects, specifically in primary cells (periodontal ligament cells) from FOP patients. This work showed how a number of differentially expressed genes might play a role in the chondrogenic and osteogenic processes, increasing the knowledge of the mechanisms by which the altered responsiveness of the mutated ACVR1 gene to Activin-A could induce heterotopic bone formation. Lyu et al. [9] have addressed the issue of inflammatory cytokines and signaling pathways, including Toll-like receptors (TLRs), involved in the genesis of heterotopic ossification in FOP, in particular, the role of MyD88-dependent pathways activated by TLRs. Using mouse models of FOP, they report that MyD88 is unexpectedly dispensable in the growth, maturation, and remodeling of heterotopic ossification lesions.

I conclude this brief editorial by reaffirming my belief that the Special Issue of *Biomedicines* on FOP has been a good opportunity to collect some of the most recent results and reports from expert researchers in the field, in the continuous effort to make progress in knowledge and new ideas. I thank all the authors who sent their contributions.
